# From Their Voices: Barriers to HIV Testing among Black Men Who Have Sex with Men Remain

**DOI:** 10.3390/healthcare3040933

**Published:** 2015-10-12

**Authors:** Thomas Alex Washington, Laura D’Anna, Nancy Meyer-Adams, C. Kevin Malotte

**Affiliations:** 1 School of Social Work, California State University, 1250 Bellflower Blvd, Long Beach, CA 90840, USA; E-Mail: nancy.meyer-adams@csulb.edu; 2 Center for Health Equity Research, California State University, Long Beach (CSULB), CA 90840, USA; E-Mails: laura.danna@csulb.edu (L.D.); kevin.malotte@csulb.edu (C.K.M.)

**Keywords:** HIV testing, social media, men’s health, health disparities, HIV, MSM

## Abstract

*Background*: HIV testing continues to be a major priority for addressing the epidemic among young Black men who have sex with men (BMSM). *Methods*: This study explored barriers to HIV testing uptake, and recommendations for motivating HIV testing uptake among Black men who have sex with men (BMSM) aged 18 to 30. BMSM (N = 36) were recruited through flyers and social media for six focus groups. *Results*: From the perspectives and experiences of young BMSM, participants recommended that information be included in HIV testing messages that would help young BMSM do self HIV-risk appraisals. Particularly, participants recommended that more knowledge about Pre-Exposure Prophylaxis (PrEP) and the role of PrEP in safer-sex practices be provided. This information is important to help those untested, or who infrequently test, better understand their risk and need for testing. Likewise, participants recommended that more information about a person being undetectable and the risk of condomless sex with an HIV negative sex partner; this information will be helpful for both the HIV negative and HIV positive sex partner for making safer sex decisions. Participants also recommended that interventions should focus on more than drug use as risk; the risk posed by the use of alcohol before and during sex deserves attention among young BMSM. *Conclusions*: These findings may inform new HIV testing interventions being tailored for young BMSM. The interventions should also consider revisiting street-based peer-outreach approaches for those young BMSM with limited access to social media campaigns due to limited access or infrequent use of social media.

## 1. Introduction

Young Black men who have sex with men (BMSM) are the group most affected by HIV in the U.S. [1]. In fact, BMSM between the ages of 18–29 represent close to half of new HIV infections among MSM within their respective racial groups [1]. Moreover, the incidence of HIV among young BMSM (aged 19–29) increased by 48% between 2006 and 2009, whereas the incidence remained stable among MSM of other racial or ethnic groups [2]. Findings from one CDC study indicated that nearly one in five (19%) MSM in 21 major US cities were infected with HIV, nearly half (44%) were unaware of their infection, and the proportion unaware was highest among BMSM (59%) [3,4]. Furthermore, previous research indicates that many sexually active BMSM do not regularly test for HIV [5].

HIV testing is a priority for addressing the epidemic among young BMSM. In fact, the Centers for Disease Control and Prevention (CDC) announced a social media campaign, “*Testing Makes Us Stronger*,” to encourage HIV testing among BMSM [6]. Knowledge of HIV status allows the individual to link to treatment. Linkage to care is important so that an HIV positive person can begin antiretroviral therapy (ART), reduce viral load, and slow or avoid the progression of the disease [7,8]. Furthermore, as the medical and behavioral science communities continue to identify best prevention practices, such as the mainstream use of Pre-Exposure Prophylaxis (PrEP), HIV testing remains a priority for primary and secondary HIV prevention efforts [9,10].

Previous research suggests that BMSM are diagnosed with AIDS later, and have less access to treatment than their White counterparts, and three year survival after an AIDS diagnosis is lower for BMSM than for White or Hispanic MSM [11]. Other studies have indicated that those who are aware of their HIV infection can be linked to the proper services for care and other assistance; proper care may help to reduce viral loads, thus making these men less infectious [12,13,14,15,16]. Additionally, men who are unaware of their HIV-positive status are more likely to engage in risky behavior, putting their HIV-negative partners at-risk for HIV-infection [17,18].

Considering the aforementioned facts, getting BMSM into HIV testing remains a crucial component for controlling the impact of HIV/AIDS among BMSM [19,20,21]. Many programs and interventions have focused on HIV testing over the decades; yet, high rates of young BMSM remain untested and unaware of their HIV status [1,2]. In an effort to encourage HIV testing uptake among BMSM, a better understanding of the barriers and challenges to testing uptake among this population, from their own perspective, is needed. Currently, research is limited in that area. However, among the few studies focused solely on BMSM, studies related to HIV testing issues and behaviors specifically for BMSM have focused on geographic setting as an influence on HIV testing [22], strategies for identifying and targeting clusters of high-risk BMSM for testing [21], and HIV-related stigma (Morris *et al*., 2014; Sayles, Wong, Kinsler, Martins, & Cunningham, 2009; Smit *et al*., 2012) and homophobia [23,24,25]. In a review of HIV stigma-related studies, Smit *et al*. [24] found some MSM avoid HIV testing due to fears of stigma and discrimination. Furthermore, Arnold, Rebchook, and Kegeles [23] found that young BMSM vulnerability to HIV was related to strategies that these young men depend on to avoid stigmatization related to homophobia from the church community, family, and within the BMSM community as well. Similarly, Scott *et al*. [26] explored the association between social support and delayed HIV testing, and their findings indicated that experience of racism and homophobia were associated with higher risk of delayed HIV testing.

Other studies have focused on HIV testing behaviors. For example, Washington, Robles, and Malotte [20] explored factors associated with HIV testing history among 102 young BMSM. The findings indicated that condomless receptive anal sex, use of the internet to seek sex, low HIV knowledge, and educational level were associated with being untested in the past 24 months. In another study, Mimiaga *et al*. [5] explored the personal and health system barriers to HIV testing among BMSM. The findings indicated that Black MSM who had a health care professional recommend HIV testing (within the last 12 months) increased the likelihood of having an HIV test. Furthermore, Hussen *et al*. [27] explored the HIV testing behaviors of BMSM. Their data revealed that BMSM’s testing behavior may be categorized by four distinct patterns: (a) regularly testing; (b) testing dependent on their relationship status or sexual behavior; (c) irregularly testing; or (d) testing avoidance. In a study regarding the willingness to use rapid home-based HIV testing (RHT), researchers found that BMSM least interested in RHT were those who reported the greatest risk-taking for HIV [28].

While the aforementioned studies have provided information important for understanding issues related to HIV testing uptake among BMSM, and despite large social media campaigns for about HIV testing, there still remain high rates of BMSM who are untested, irregularly test, and who are HIV positive and unaware of their HIV status [1,2] . These facts emphasize the need to better understand barriers and challenges for those who are not regularly testing, particularly from their perspective. The goal of this article was to better understand the perspectives and experiences of young Black MSM aged 18 to 30 regarding HIV testing uptake. More specifically, we explored the barriers and challenges to HIV testing uptake behavior from the perspectives and experiences of young BMSM.

## 2. Methods

### 2.1. Participants

A total of 36 young BMSM participated in six focus groups. Because we wanted to understand the perspectives of young BMSM whose status is unknown and have not tested within the last 24 months; tested positive in the last 24 months; and tested negative with the last 24 months, we recruited using those criteria and placed participants in groups based on the group criteria (see Table 1). The additional sample criteria included: Black/African American male, aged 18 to 30 (median age = 23), resident of Los Angeles County, sexually active with another male in the last six months, and unprotected sex at least once in the last six months. Participants were recruited through local magazine and internet (Facebook^©^, Menlo Park, CA, USA, Myspace LLC) advertisements; palm cards distributed at local colleges and universities; flyers distributed outside gay establishments and hetero-normative clubs that were frequented by a mixed crowd of heterosexual, bisexual, and homosexual patrons; gay, lesbian, bisexual, and transgender (GLBT) clubs and lounges; and organized events, such as Gay Pride Festivals.

**Table 1:** Characteristics of participants by focus group assignment (N = 36).

Group Criteria	Sub-Sample Size	Number of Focus Groups
Unknown status or Untested in last 24 months	*n* = 14	2 Groups
Tested positive within last 24 months (self-reported)	*n* = 11	2 Groups
Tested negative within last 24 month (self-reported)	*n* = 11	2 Groups

### 2.2. Procedure

The institutional review board of the principal investigator’s home institution approved the study procedures. This study integrated two major components. First, a self-report paper/pencil survey was administered to the participants to assess sociodemographic characteristics, HIV prevention knowledge, HIV prevention communication knowledge, safer-sex practices, drug using behavior, HIV testing history, sexual behavior, and general health motivation. The measurement properties used can be found elsewhere [20] Additionally, informed by our preliminary findings showing reports of high levels of risky sex and alcohol use, a string variable, “Drinking settings,” was included on the questionnaire. Respondents were asked “*In the last three months*, *list the type of place where you most consumed alcohol (*e.g., *bar/club*, *home*, *car)*”. We then created categories from the lists, and grouped these by majority themes reported. The resulting categories were as follows: Bars/Clubs; Home drinking (home, buddy home (friends home), neighbors house); Car (e.g., reporting “car,” “garage,” or “car wash”); Public spaces (park, alley, *etc*.).

Second, qualitative data were gathered through semi-structured focus groups. Focus groups are an effective means to evaluate services or test new ideas, and are an excellent way of involving community members in developing an intervention [29,30,31]. Each focus group session included approximately 5 to 7 participants and lasted approximately 90 min. Focus groups were convened at the affiliated university and a community-based organization easily accessible by public transportation, and available parking. Sessions were audio-recorded. Participants were compensated $ 50.

Participants responded to questions regarding barriers and challenges to HIV testing uptake, such as: What are some reasons you or your sex-partner may have taken an HIV test, but didn’t return for the results? What are some reasons you feel your peers may not get tested for HIV? If you were seeking HIV testing, from whom would you prefer to receive the test? Focus group questions were similar, but varied slightly to reflect each group’s criteria. For example, in the group of BMSM who had not tested within the last 24 months were asked, “*What are some reasons you have not tested in the last 24 months?*” Whereas, in the group of BMSM who had tested positive in the last 24 months, they were asked, “*When you last tested for HIV*, *what motivated you to seek HIV testing?*” Again, the overall goal was to learn from each group, barriers and challenges to HIV testing uptake, whether it was why one group hasn’t tested, and what motivated the groups that have tested to uptake testing. Participants were also asked to provide recommendations to include in an intervention to encourage HIV testing.

### 2.3. Coding and Analysis

Survey data: Frequency distributions and crosstabulations were computed to describe characteristics about the sample (see Table 2).

**Table 2:** Demographic Variables for Study Participants (N = 36).

Variable	Percent (n)	
Age		
<Mean Age 23	53 (19)	
≥Mean Age 23	47 (17)	
Educational Level		
High School or less	64 (23)	
At least Some College	36 (13)	
Employment Status		
No Employment	36 (13)	
Part/Fulltime Employment	64 (23)	
Had HIV Test past 2 yrs		
No	39 (14)	
Yes	61 (22)	
Receptive Anal Sex Risk **		
No	47 (17)	
Yes	53 (19)	
Insertive Anal Sex Risk **		
No	61 (22)	
Yes	39 (14)	
Seek Sex on the Internet		
No	28 (10)	
Yes	72 (26)	
Drug Use Risky Sex **		
No	30 (11)	
Yes	70 (25)	
Alcohol Use Risky Sex **		
No	17 (04)	
Yes	83 (20)	
Drinking Settings *		
Clubs/Bars	40 (14)	
Home	75 (27)	
Car	83 (29)	
Public	60 (22)	
HIV Related Stigma		
Low	36 (13)	
High	64 (23)	
HIV Knowledge		
Low	47 (17)	
High	53 (19)	

** Condomless sex in last three months; * Participants were allowed to list multiple drinking settings.

Focus group data: The audio recorded focus group interviews were transcribed by a professional transcription service. Transcripts from each of the three types of focus groups were reviewed with the goal to identify the common themes from each group type and determine the similarities and differences across groups. To that end, two trained qualitative researchers coded the data by repeatedly reviewing the participants’ recorded responses (*i*.*e*., transcripts), and several categories were identified. Central to the procedures of qualitative research are the selection of a core category and relating all major categories both to it and to each other [29]. After identifying major categories, process notes were developed, participants’ responses were compared using Cohen’s kappa, and central themes relayed through the participants’ responses were identified. Cohen’s kappa was used to explore a significant measure of agreement for the degree to which the two reviewers’ codes were applied to the data. The goal was to allow patterns and common issues shared among the participants concerning issues and barriers to HIV testing and HIV-status awareness among sexually active unknown-status Black MSM. This method of “emerging” analysis is referred to as the naturalistic inquiry or constructivist paradigm.

## 3. Results

### 3.1. Background Characteristics of the Sample

As shown in Table 2, the self-report questionnaire revealed that the mean age was 23 years, 53% of the participants were aged less than the mean age 23, while 47% were aged 23 or greater. The majority of the participants had high school or less education (64%), and were employed at least part-time (64%). Approximately, 40% of the participants were untested for HIV within the last two years. More than half (53%) had receptive anal sex without a condom, and 39% had insertive anal sex without a condom in the preceding 3-months. The majority of the participants used the Internet to seek sex in the preceding 3-months (72%). Seventy percent of the participants had condomless sex when using illegal drugs and 83% had condomless sex when using alcohol. Participants reported drinking alcohol in the following settings: clubs/bars (40%), home (75%), car (83%), and public (60%). Approximately 64% reported a high level of HIV-related stigma, and 53% had high HIV knowledge.

### 3.2. Barriers to HIV Testing Uptake from the Voices of BMSM

After review of the transcripts, respondents’ comments were organized using five categories that BMSM perceive as barriers to HIV testing uptake: (1) lack of knowledge for conducting a self HIV risk appraisal; (2) anxiety and substance use; (3) lack of peer support for testing; (4) stigma; and (5) perceptions about HIV testing and treatment facilities.

### 3.3. Lack of Knowledge for Self HIV Risk Appraisal

Responses revealed that many of the participants had a lack of knowledge about HIV and HIV risk behaviors and practices. It was commonly stated across all groups that more knowledge is needed about HIV. Participants expressed that they get confused about whether a person who is the top [inserter], who does not use a condom during sex, can get HIV. Another common concern was whether it is safe to have condomless sex with a person who states he is undetectable. Additionally, participants expressed concern about risk of HIV and the need to test for HIV if condomless sex was performed while on PrEP. A participant stated:

“*I need to know more about HIV in general…like if a dude is undetectable—what does that mean exactly? Does that mean I can have fun with him*, *do it with no cover [condom]? How can I tell if a guy is sick [HIV positive] that I did it with…so I’ll know if I need to get tested?*”

A second participant stated:

“*How do I know when I should get tested? I’m not sure if what I do…I think what I do is okay [safe] and no need to be worried*. *But*, *then again*, *I’m not really sure*.”

“*Do the pills they have for negative people mean they can do it free [have condomless sex] and keep them from getting it [HIV]…why do they need to test [for HIV]?*”

### 3.4. Anxiety and Substance Use

Responses revealed that a major barrier to HIV testing uptake was anxiety about receiving an HIV positive test result. Participants in all groups emphasized that for themselves, and for their friends, they believe the hesitance was the fear of learning that they might be HIV positive and were not ready to deal with it. This finding was similar for the majority of those in the group untested for HIV within the past 24 months, those who had tested and self-reported a negative HIV status, and those who were HIV positive. The HIV positive group discussed that they had anxiety about testing, and most were motivated to test after having had a friend or sex-partner disclose that they tested positive for HIV. A participant from the group of men who were untested for HIV in the past 24 months, stated:

“*Fear of what happens if I get a positive result…will I be able to manage… anxiety about the possibility of if I have a positive result should I get tested*.”

Another participant stated:

“*…I’m afraid to get tested ‘cause I know I been doing some stuff that ain’t safe*, *and I might be [HIV] positive*. *I don’t know how I will handle it if I found out…I ain’t got no money for the medicine and going to the doctors*.”

An auxiliary finding related to anxiety about receiving an HIV positive result was that many of the men reported engaging in other behaviors that might be related to risky sexual practices, such as using alcohol and/or drugs before or during condomless sex. Substance use with sex was a commonly reported issue. A participant stated:

“*Knowing I sometimes hit the booze*, *smoke weed or blow clouds [meth]*, *and ain’t use no condom from time to time make me afraid that I might have got it [HIV]*, *so sort of afraid to test*.”

Another participant stated:

“*…me too*, *[laughter]*, *sometimes freak out too and scared to get tested…when I think about it…especially cause I been fuckin with 4–20 [marijuana] and drinkin*. *I don’t always use something [condom]*, *just fuck without it…but I pull out*, *but still freak out later when I know I should get tested*.”

### 3.5. Lack of Peer Support

Participants suggested that a barrier to HIV testing uptake was the lack of peer support. The majority of the participants stated that they don’t talk much about HIV testing with their friends, or encourage friends to test for HIV. However, a few of those who tested stated that they tested with a friend. Among the group who are HIV positive, the most common testing motivation was having had a friend or sex-partner disclose that they tested positive for HIV. Participants from all groups emphasized the importance of having peer support. One participant stated:

“*I’ve been out with friends and we see the big white van [HIV testing van]*, *but it’s funny cause it’s like the big elephant in the room…walk on by it many times*. *If one of them would say let’s stop and do it [test]*, *I would so go for it*.”

Another participant stated:

“*Man*, *it would be cool to have somebody to go [get tested] with*. *We never talk about it though*. *Only thing we say stuff like who you sleeping with*, *or say I heard he got it [HIV]*. *That dude got the bug [HIV]*. *Never say you should get tested*, *or go with me [to test]*.”

### 3.6. Stigma

Participants discussed contextual barriers to HIV testing uptake that influence young BMSM. The most commonly discussed barrier was stigma, which reflected concerns about being judged as a person with HIV. Those in all the groups expressed the negative messages they hear from friends and family about people who are HIV positive. Participants in all groups discussed their experiences being in settings where people “said ugly or nasty things about people having HIV.” One participant, from the HIV positive group, described a situation he faced among a group of friends:

“*…was with a group of friends I usually go hang out with*, *like we go to the clubs*, *play ball*, *and stuff together*. *We were driving to [club name removed]*, *my friend [A] said he found out that the dude he was fooling with had HIV*. *My other buddy [B] said*, “*I hope you ended it right then*.” *So*, *my buddy [A]*, *said hell yeah*, *ain’t got time; I ain’t gonna be getting sick and shit*. *They don’t know I got HIV…I was quiet…didn’t know what to do…what they think about me if I told it*.”

A participant from the untested group stated:

“*If I got positive [for HIV]*, *then*, *if my family found out*, *I would be so ashamed that they know*. *My mama already think all gay people get HIV from being in the gay lifestyle*.”

### 3.7. Perceptions about HIV Testing and Treatment Facilities

Participants reported concern about the physical location of the HIV testing unit within medical/clinic facilities, and the location where testing vans are located. They perceived that there was very little privacy, or in many cases, the testing unit was located in a space where it was obvious that one would be going to take an HIV test. Participants stated that they and many of their peers may be ashamed to seek testing within a setting where the HIV testing unit is not private or ambiguous. Similarly, participants across all groups had the same concern about facilities where one could receive HIV treatment. Those receiving treatment discussed their preference to attend a private doctor, or a treatment facility that is not readily identifiable as an HIV treatment facility. An HIV positive participant stated:

“*I know people wonder*, *just like I used to wonder*, *if I take the test and positive*, *what type of place will I go for treatment…people will know why I’m there…I don’t have no way to go to a private docs*. *But*, *the place I actually go to is a nice clinic…they see only people with HIV—it’s part of a hospital*, *but got its own area…not as bad now*. *But I did used to wonder*.”

An untested group participant stated:

“*Man*, *the [HIV] testing van is out in the open*. *Shoot*, *if I get a bad [positive] result*, *where I can go*. *Man I be out on way to have fun—that won’t be nowhere to go to be private if I got bad news like that*.”

## 4. Discussion

HIV testing continues to be a critical component to HIV prevention efforts, and many strategies have been implemented to increase testing. Yet, there still remain high rates of BMSM who are untested, irregularly test, and who are HIV positive and unaware of their HIV status [1,2]. The study findings identified current barriers to HIV testing, and revealed barriers that still remain after three decades of providing HIV prevention campaigns and interventions. The findings provide a better understanding from the perspectives and experiences of young Black MSM aged 18 to 30 regarding HIV testing uptake, and recommendations that may be helpful for addressing these barriers moving forward.

First, the findings suggest that young BMSM are lacking knowledge that is important for doing self-appraisals about their risk for HIV, and the need for testing. These young people have been inundated with much information in recent years. For example, the medical and public health communities have promoted treatment as prevention suggesting that an HIV-positive person can achieve undetectable levels after undergoing and remaining compliant with antiretroviral therapy (ART), and thus an undetectable viral load reduces the likelihood of HIV transmission [32]. Moreover, in the more recent introduction of PrEP for those who are HIV negative, young BMSM are receiving mixed messages about PrEP and its effectiveness [33]. It appears that the information about the importance of consistent condom use is unclear or lost in the message concerning these prevention strategies. Better information on the specifics of HIV as it is today, including the meaning of being undetectable, the usage of PrEP, and the risk of condomless sex, should be provided to young BMSM, regardless of HIV status. This information is critical for young BMSM to understand the need for consistent HIV testing and HIV treatment.

Second, the findings revealed that young BMSM are drinking alcohol before and during sex, and as a result having condomless sex; thus, increasing their risk for HIV. For many of these young BMSM, they noted that they think more about drug use and sex risk than they did about alcohol use and sex risk. Recent research have shown that alcohol use and sex risk exist among young BMSM [18,34], yet, HIV prevention and testing interventions focus more heavily on drug use and sex risk. HIV testing interventions are needed for young BMSM that focus on the risk of alcohol use before and during sex.

Participants suggested the need for peers to be more proactive with encouraging their social networks to discuss HIV, and consider HIV testing together. A campaign or intervention that includes a peer-based approach was recommended. However, the participants recommended that interventions using social media should still include a street-based peer-outreach component; young BMSM at high risk for HIV may have limited access or inconsistent use of social media. Peer-based approaches have been useful for influencing health behaviors among many subgroups, including young minority men who have sex with men [35,36,37,38,39,40,41]. Similarly, street-based approaches have been successfully used most often to influence health behaviors among sex workers and injection drug users [42,43]. To collectively address a barrier that continues to remain—anxiety about receiving an HIV positive test result, stigma, and negative perceptions about HIV testing facilities—the participants recommended providing more information about “*what it means to be positive today*.” Participants recommended that the material be offered as part of HIV prevention messages. There are campaign websites that provide information targeting BMSM regarding the HIV Continuum of Care, such as “Positive Spin”. Positive Spin was created as part of The HIV Care Continuum Initiative of the National HIV/AIDS continuum [44]. The website provides a series of real stories from individuals sharing their experience along the HIV Continuum of Care. Additionally, the website provides resources for locating an HIV testing site. This is a great resource for those who become aware of the website. However, peer outreach through social media networking and street-based outreach are critical to linking and engaging young BMSM along the continuum of care. Participants recommended that “some sort” of intervention be developed to address these pressing issues, and the material should be available to people who have not tested, or those considering uptake of HIV testing. Furthermore, the findings indicate that peer support is an important issue to consider for encouraging HIV testing among young BMSM. BMSM from all three subgroups in the study, HIV positive, HIV negative, and untested within the past 24 months, reported on how a friend may influence whether or not one tests for HIV. Those who had tested emphasized that having a friend or sex partner who was HIV positive, or having a friend accompany them for testing, was a motivating factor for testing uptake. Peer support has shown to be an important component for encouraging health behaviors among peers [22,26].

Findings from this study reiterate the need to identify ways to address stigma in an effort to encourage HIV testing uptake. HIV stigma (labeling, stereotyping, status loss and discrimination within a power structure) [45] is germane to the HIV epidemic in MSM communities and is associated with adverse effects on both HIV testing behavior and linkage to care [20,23,24,25,46]. For young MSM, in particular, HIV stigma is related to depression and low self-esteem and may lead to concerns about status disclosure [47], and associated with increased rates of unprotected sex while under the influence of a substance [48].

BMSM also wanted more information about the location, and climate and culture of HIV testing and HIV treatment facilities. BMSM identified lack of knowledge on, and having negative perceptions of, HIV testing facilities, as a barrier. Knowledge of testing facilities has been identified in other research. For example, in a study of young BMSM, Mashburn *et al*. [22] found that the strongest influence on HIV testing across three U.S. geographical locations was knowledge of a comfortable place for an HIV test. In the current study, BMSM recommended to make HIV home test kits available and more affordable to address the stigma and negative perceptions about HIV testing facilities. Making HIV home test kits affordable and more accessible may be worth exploring. In fact, Young *et al*. [49] found that young Black and Latino MSM considered that a vending machine was an acceptable HIV test delivery method. Further research is needed to explore the issue of cost as a barrier.

There are several limitations to this focus group study. The BMSM were self-selected and may not be representative of all BMSM. Sampling methods of the participants also may have led to a selection bias. Some of the participants in the groups may have known one another from working in similar settings within the BMSM community. There is a possibility that the BMSM in the different groups may have known each other due to convenience sampling. Knowing others in the groups may have lessened the openness of participants’ responses. Also, a majority of the BMSM had a low level of education (high school or less education), and were substance users; thus, they may not be representative of the BMSM community. Socioeconomic status was not assessed. Yet, it is an important factor that should be considered in future studies. It would be of interest to explore whether these findings are similar or differ among BMSM across socioeconomic strata. Lastly, the participants were from three different subgroups of BMSM, HIV positive, HIV negative, and untested within the last 24 months; as such, we recognize that their experiences and insight may vary between and within the groups, but also may not be representative of the subgroups of BMSM. Notwithstanding these limitations, our findings are worthy of consideration to inform new interventions aimed at encouraging HIV testing uptake among young BMSM. The findings suggest the need for interventions to not only focus on the HIV Continuum of Care, but the need to include multiple platforms for delivery, including social media, peer-based and street-based approaches. These platforms are necessary for reaching young BMSM. The findings offer perspectives of young BMSM regarding the need for better awareness and education about the role of PrEP and HIV prevention, and improved health literacy to educate about medical jargon that can sometimes be confusing or overwhelming for lay persons.

## 5. Conclusions

As previously mentioned, over the past 30 years, many strategies have been implemented to increase HIV testing. Yet, new and past barriers to HIV testing still remain. There continue to be high rates of BMSM who are untested, irregularly test, and who are HIV positive and unaware of their HIV status [1,2]. HIV testing interventions should not just highlight the importance of testing, but should emphasize the continuum of care. As new advances among the HIV continuum occur, up to date interventions addressing health literacy are crucial. Health literacy should consider young BMSM’s capacity to obtain, process, and understand basic HIV health information in an effort to make appropriate decisions about their health [50,51,52,53,54]. HIV testing programs should be inclusive by providing knowledge about PrEP and consistent condom use for those who are HIV negative, and linkage to care for those who test HIV positive. The interventions should provide knowledge about “*what it means to be undetectable*”. The information should be written in a way so that both those who are HIV negative, and HIV positive, can make informed decisions about condomless sex. More too, the information should be written so that young BMSM will be able to do self HIV-risk appraisals and better understand the importance of regular HIV testing. HIV testing interventions focused on alcohol use and condomless sex as risk are needed. Lastly, the use of social media continues to grow in popularity and is used to deliver public health campaigns. However, HIV testing interventions should use both social media and street-based peer-outreach to reach young BMSM, particularly for those who may be better reached through grassroots methods.
